# Improvisation and meaning

**DOI:** 10.3402/qhw.v8i0.20604

**Published:** 2013-08-07

**Authors:** Simon Gilbertson

**Affiliations:** The Grieg Academy, Institute of Music, GAMUT- Grieg Academy Music Therapy Research Centre, University of Bergen, Bergen, Norway

**Keywords:** Improvisation, music therapy, arts-based research, traumatic brain injury, long-term repeated-immersion, case study, therapeutic narrative analysis, musicological analysis

## Abstract

This article presents and discusses a long-term repeated-immersion research process that explores meaning allocated to an episode of 50 seconds of music improvisation in early neurosurgical rehabilitation by a teenage boy with severe traumatic brain injury and his music therapist. The process began with the original therapy session in August 1994 and extends to the current time of writing in 2013. A diverse selection of qualitative research methods were used during a repeated immersion and engagement with the selected episodes. The multiple methods used in this enquiry include therapeutic narrative analysis and musicological and video analysis during my doctoral research between 2002 and 2004, arts-based research in 2008 using expressive writing, and arts-based research in 2012 based on the creation of a body cast of my right hand as I used it to play the first note of my music improvising in the original therapy episode, which is accompanied by reflective journaling. The casting of my hand was done to explore and reconsider the role of my own body as an embodied and integral, but originally hidden, part of the therapy process. Put together, these investigations explore the potential meanings of the episode of music improvisation in therapy in an innovative and imaginative way. However, this article does not aim at this stage to present a model or theory for neurorehabilitation but offers an example of how a combination of diverse qualitative methods over an extended period of time can be instrumental in gaining innovative and rich insights into initially hidden perspectives on health, well-being, and human relating.

On any one day, in any place on the planet where motorization has spread, there is the risk that a healthy member of society might be torn from the very material and meaning of their lives and the lives of those around them as a result of a road traffic incident. Worldwide, road traffic crashes have been estimated to be responsible for approximately 1.2 million deaths and up to 50 million injuries per year (World Health Organization, 2004). Although the World Health Organization (2004) describes the relative risk of serious injury during each single journey as being low, every year there is an increase in the total number of survivors of road traffic crashes living around the world. In terms of severity, survivors of road traffic crashes who experience a traumatic brain injury experience a sudden and dramatic loss of health and well-being. These catastrophic events often occur before the very eyes of those around them, and “for people who have experienced traumatic brain injury, meaning and sense in life may become disrupted, distorted and, for some, hidden” (Gilbertson & Aldridge, 2008, pp. 11, 12).

The National Institute for Health (NIH, 1999) has provided a sequence of descriptions of the effects of traumatic brain injury in which, first, the neurological consequences of how the brain may be damaged are described, and, second, a list of concepts is given concerning how the site of brain damage will negatively affect the performance of movement, cognition, speech, and social interaction. In the same document, the NIH (1999) provides a list of therapies, including music therapy, with information regarding the level of evidence of effect of the treatment for the consequences of the traumatic brain injury. Importantly, other commentators (Agrawal, 2012; Kreutzer et al., 2009; Perlesz, Kinsella, & Crowe, 1999) have highlighted the need for an additional consideration of the effects of traumatic brain injury on the lives of the injured individual's closest family.

The literature on music therapy and traumatic brain injury has grown during the past decades to include guidelines for therapists, a Cochrane review and literature reviews on children and adults (Baker & Tamplin, 2006; Bradt et al., 2010, Gilbertson 1999; 2005a; 2008; 2009). But how, then, can qualitative research aid in identify the meaning of therapeutic strategies, such as, in this particular case, music therapy? This is the question addressed in the remainder of this article, which presents and discusses how multiple qualitative research methods can be created and applied to generate different understandings and perspectives from a single case of study. By considering one case of a young boy with severe traumatic brain injury in this manner, this article offers an example of how long-term repeated-immersion in research can be used to identify significant aspects of the process of rehabilitation.

## The case: Episode 1

In August 1994, a 15-year-old boy was referred to individual music therapy in the early neurosurgical clinic in Germany where I worked as one of a small group of music therapists. The teenager, who I will call “Bert,” had been involved in a serious road traffic incident in which a car had crashed into him as he was riding his bicycle. The incident caused a multitude of injuries, including a severe traumatic brain injury and fractures of his skull and both legs. As a result of the initial injuries, Bert presented extreme levels of increased muscle tone, and he did not have any spontaneous speech, verbal gestures, or capacities of conventional physical gestures. He did not follow movement with his eyes, nor was it possible to effectively determine the ways in which he might or might not perceive the environment and those around him.

On 11 August, Bert's mother brought him in his wheelchair to one of the clinic's music therapy rooms for his first individual music therapy session with me.

The events of that music therapy session would turn out to be the subject of many years of reflection and research. In particular, there was one episode of interaction lasting 50 seconds in which the young boy moved his left leg, which created sounds from a percussion instrument called a wind chime, while I was improvising on the piano. This motion occurred at a time at which this same leg movement had been observed outside of the music therapy setting, but had not convinced any observers that it was of a communicative nature and was considered within the multidisciplinary team meetings to be without any meaning, simply being a meaningless result of neural activity. Contrastingly, in that music therapy session I had experienced a tentative sense of some form of connectedness with Bert, but could not at that time clearly identify the exact nature or content of this experience.

A consideration of an individual's ability to improvise sounds and music with the music therapist allows for the expression and perception of communicative potential even at times when a full realization of this potential is not conventionally considered. Though it is beyond the scope and intention of this current article, a full portrayal and discussion of the improvisational approach to working with Bert and other individuals in early neurosurgical rehabilitation are presented elsewhere (Gilbertson & Aldridge, 2008). Whereby this process of improvisation may require a phase of exploration and adjustment on the part of the therapist, co-improvised music making becomes an arena of interpersonal exchange. This may apply no matter how unconventional or idiosyncratic it may be in comparison to conventional and integrated actions and interactions, as portrayed in the following descriptions of events in music therapy in early neurorehabilitation:

Single, breathy, vocal utterances have completed cadences and led to a sharing of melodic phrases. Finger movements limited by spastic muscle patterns and so fine that they must be described in millimetres have determined the direction of musical improvisations and dialogues. These fine, often minute, movements and vocal sounds have sometimes developed into a repertoire of physical and communicative gestures that can form the basis of developing relationships in the context of shared musical activities. Gigantic, explosive explorations of steel drums, gongs and large drums have contrasted the stillness of patients no longer able to speak. (Gilbertson & Aldridge, 2008, p. 13)

In the subsequent years after 1994, the music therapy department in the clinic grew to become the workplace for seven full-time music therapists. Music therapy became established as an integrated and integral element in the multidisciplinary approach to early neurosurgical rehabilitation. In those years, I returned to the episode many times for reflection and viewing with colleagues from various professions. I used the video recording of the episode in national and international presentations to provide an example of the work we were doing at the clinic. Through a pragmatic process of archiving, only those recordings of episodes of therapy considered to be significant and representative for our practice and this particular episode were archived.

## Episode 1: A new starting point in a doctoral research project

In 2002, I began my doctoral research on music improvisation with people with severe traumatic brain injury (Gilbertson, 2005b). In the study, I chose to carry out a retrospective explorative study to find out if it was possible to identify any links between the elements of music improvisation and clinical change during the rehabilitation process. To undertake this exploration, I selected the method of therapeutic narrative analysis (Aldridge & Aldridge, 2002), a form of qualitative research based on personal construct theory (Kelly, 1955). Within that framework, I included musicological and video analysis in the research process. After reviewing many tens of hours of video and audio archives of music therapy sessions that I had carried out during the previous 8 years, I selected “Episode 1,” from 11 August with Bert, as one of 12 episodes for closer examination (Gilbertson & Aldridge, 2008).

Episode 1 represented a specific situation representative of many processes of recovery following severe traumatic brain injury. In the episode, the extreme level of isolation and idiosyncratic actions of the person with traumatic brain injury creates a high level of interpersonal disorientation and secondary isolation of the person they are currently with, in this case the music therapist. Within the therapeutic narrative analysis, I created and included various sources of data: a musicological transcription and analysis, a sonograph of the frequencies and dynamic qualities of the episode, alongside a narrative text based on actual clinical notes made at the time of the therapy process. It is from this episode that the significance of the role and nature of isolation and minimal contact in early neurorehabilitation was elicited. This is a significant aspect of early phases of rehabilitation in regard to actions made by severely traumatically injured patients, particularly as these actions are commonly at risk of initially being interpreted as being without meaning or consequence.

In the episode, which I labeled “First foot forward” during the research process, I improvised a four-phrase melody beginning on the note “f” with my right hand, which was accompanied by simple, open-chord structures in my left hand in the tonality of “F” major (for a full transcription of the episode, please refer to Gilbertson & Aldridge, 2008). Using video analysis, it became evident that there were possible tentative links between the timing of my improvising on the piano with Bert's mouth movements and leg movement at the close of the melodic phrase and cadence. The narrative analysis of my therapy notes showed that a form of initial, but uncertain, synchronization was being attempted, although my notes also showed that I was not certain at the time whether Bert was aware of the potential link or not. Interestingly, in the process of peer checking the validity of my video transcription and repeated viewing with colleagues and my research supervisor David Aldridge, clear signs of changes in my improvising were commented upon. It seemed that the movements and actions that Bert was making were leading and directing aspects of my actions. It became clear that, particularly during this initial phase of isolation following severe traumatic brain injury, I was changing and adapting my own musical behavior in minute detail and relation to the minimal movements and sounds made by Bert. Thus, by allowing myself to be led to improvise music based on Bert's actions, it was possible to gain a perspective from which it is possible to perceive myself in relation to Bert. A relational description of the quality of isolation became possible through identifying links between real-time music improvising. Through analysis and synthesis of constructs and core themes of the further 11 episodes, I selected the superordinate category pairs of *isolation* and *integration*, and *idiosyncrasy* and *convention* to represent the findings at higher levels of abstraction. These superordinate concepts led to the elicitation of the core category of *relation(ship)* and its significance in the process of rehabilitation following severe traumatic brain injury (Gilbertson & Aldridge 2008).

### A second perspective: expressive writing to explore a reframing of the episode

In 2007, I moved from Germany to Ireland to work at the Irish World Academy of Music and Dance, University of Limerick. There, I worked together with Professor Jane Edwards and Dr Alison Ledger at the Master of Music Therapy training course. With the episode still in my consciousness, my new teaching role and the curriculum designed by Jane Edwards brought me into contact with ecological and relational perspectives informed by the work of authors, including Bronfenbrenner (1979), Vygotsky (1978), Salter Ainsworth and Bowlby (1991), and Schore (2000). At exactly the same time, I began working with David Aldridge on adapting the manuscript of my doctoral research into a book format (Gilbertson & Aldridge, 2008). During 2007–2008, I had become aware of how my understandings of the original Episode 1 from 1994 had become reframed and recontextualized through these curriculum readings. I became starkly aware of the need to reconsider the episode in terms of Bert, his family, and indeed the nature of my own decisions as a therapist in 1994.

During this time of reconsideration, it was necessary for me to contact Bert's mother in relation to requesting permission to publish the details of the therapy process in the planned book. During written exchanges with Bert's mother, she wrote to me that she was never really sure what had gone on in the music therapy room, but that she had always believed that it was something good for her son. Rather than experiencing this potentially as flattery, I was deeply disturbed by the fact that it had taken me nearly 14 years to find a theoretical stance from which I could consider the episode in light of the needs of the families of those injured by traumatic brain injury. It felt as if a very significant new aspect of the episode was emerging. I decided to explore this situation in a very raw and direct manner and engaged in expressive writing about the episode and my experiences of the therapy process with Bert. This choice had been sparked by conversations with my colleague Jane Edwards and also my colleague Alison Ledger's use of arts-based research methods, including poetry writing, in her then ongoing doctoral research (Ledger & Edwards, 2011). In one of the various pieces of expressive writing, I explored my reflections in relation to Bert, his mother, and my actions in a fictitious letter to Bert's mother. Before drafting the actual letter, I first described the moment of engaging with the writing process as follows:

As I write in this moment, I sense my breath leaning heavily outwards onto my ribs and feel my chest filling slowly and deeply. The time has come to write an apology, from a space that is open and authentic, but also respectful and timid.

The letter, which appears below, was not written to be sent to Bert's mother, but to explore and verbalize new perspectives through the creative writing research process:

Dear Mrs. D.,

After your son was admitted to the rehabilitation clinic, he received music therapy sessions with me four times a week. As I am certain you will remember, each session lasted somewhere between 15 and 20 minutes depending on how things were going on that day. Your son was in a non-life-threatening but serious health situation as I met him for the first time. His arms and legs were contorted and twisted by the high level of muscle tone in his body and the scars on his shaven head caused by injury and surgery were clearly visible.

His body-sweat and drooling left him permanently soaked and towels were drenched within minutes. His gaze, unchanging, gave nothing away to us about his actual experience of his life. You had bought him some stuffed toys to support his arms and to stop the plastic of his wheelchair table digging into his skin. He was in good hands.

During the 20 minutes he was in the music therapy room, you sat outside, alone. Though it is many years ago, I remember there were two chairs outside of each therapy room in the corridor. They were put there extra for Mum, Dad, daughters and sons, brothers and sisters, grandmas and grandpas, and everyone else who was visiting.

The music therapy room was well sound insulated. The architects had been well informed, taking advice from a music therapist whilst drawing up the plans. A small amount of noise leakage is inevitable in any construction like this. After all, it is a hospital and not a famous recording studio.

I imagine that the sounds ‘leaking’ from the therapy room must have been unusual. Your son was severely injured, not long since surviving a horrendous road traffic incident. Now, someone was singing for your son, playing music on the piano.

I know we spoke with each other after every single session. As I steered your son carefully in his oversized wheelchair through the doorway, you would ask, ‘How did it go? How was he today?’ and I would tell you what I had done, singing to the rhythm of his breath, opening to any signs of communication. … It was often hard to find words to describe the immeasurable changes that I thought I had perceived, or not. Not many people thought I would ever have anything to report. But we carried on meeting undeterred. You stayed outside patiently waiting.

One day he moved his leg which caused the wind chimes to sound right at a point in the improvised music that it sounded like a film score, arranged to the perfect moment. No professional composer would have been able to do it better. You would have loved it. I wish I had been ‘awake’ enough to let you in.

Another day, he started to play the grand piano. Yes, it was unconventional – it was with the big toe of his right foot – but it was great. He plonked around on the high keys while I played some heavily rhythmic stuff down at the lower end of the piano keyboard. It would have been great for you to see it. Particularly as things were so difficult at that time, with all the rest of life.

There were so many days that you would have enjoyed seeing his progress. Or even joined in. Maybe it would have helped? I'll never know.

After a very long time, your son was doing really fine and ended his individual music therapy sessions with me. Together with a colleague we decided it would be good to start up a new music therapy group, and your son should be one of the first members. The group was quickly arranged and I remember you came in. You stayed for the whole session sitting next to your son. The memory of your smiles still brings me to tears. You seemed so proud, so full of something essential to life, something that rehabilitation is all about. You didn't say much. Here and there, a little ‘well done’, a quiet ‘great!’ and a touch as light as a feather on his arm after he had played his musical instrument together with the others. You went completely red in the face as the therapist asked if you would like to have a go, joking the offer off with a huge ‘What me? I can't play for my life!’

I know I'm rambling on a bit, but what I really wanted to say was, I'm sorry.

I'm sorry I didn't recognise that you were missing – missing from the room and from the opportunity to use music, just as I had done, to reduce the isolation and trauma caused to you and your son by the traumatic injury to his brain.

By not entering the music therapy space, it was you who had made it possible for me to enter into such an intimate microcosm with your son. Of course, there are things that I can do with music. If it is possible, as research has shown, that music can be used in therapy to reduce isolation and idiosyncratic situations, and to increase integration and the use of conventions, then I must apologise that you were missing from this situation.

I am grateful to you for providing a frame of trust as a mother for your son, a young man with traumatic brain injury, and for me, the music therapist I was back then.

Through this reframing and recontextualization of Episode 1, which was initiated by new reading and the expressive writing process, it was possible to express a hidden realization of the importance of the interpersonal and social contextualization of the individual with traumatic brain injury and the people affected by the injury, in this case Bert's mother. The self-immersed quality of the letter is as striking as the heavy emphasis of gratefulness and my use of “I” whilst articulating the fragility of the therapist's role. Alongside domains of relational aspects that were hidden in the first research project, the letter also communicates an impression of the naïveté of an early-stage therapist. The session from which Episode 1 is taken took place approximately 1 year after I completed my formal training as a music therapist. It may be that the content and expressions used in the letter overemphasize the significance of the therapist, but more importantly though than considering the therapist alone, the letter aids in recognizing the role of active relating for the patient and his or her family members. Rather than considering this aspect as an auxiliary or solely complementary perspective in rehabilitation, the central importance of the meaning of music therapy in rehabilitation processes in terms of the social nature of the traumatic injury became clearer and could be verbalized. Many years after the original episode, engaging in expressive writing as an additional methodological strategy added an additional layer of understanding and reflection on Episode 1. The verbalization of the social nature of traumatic brain injury pointed toward the significance of a social response in the rehabilitation process. This hinted strongly toward the need for a consideration of a model of music therapy in the early neurosurgical rehabilitation that is based on social, and not individualized, conceptualizations of function, cognition, capabilities, and agency in future research. Rather than looking solely at issues of how therapy can enhance individual brain plasticity, this work suggests the relevance of a consideration of the concept of *social plasticity* that acknowledges the unavoidable nature of both constructive and destructive change as collective and relational.

### A new direction in qualitative arts-based research on Episode 1

When reading my clinical notes on Episode 1 during the past 19 years, I have repeatedly been struck by the languaging and coding of the narrative reports of my experience. One such comment caught my attention: “It would be great to find a way of getting in touch with each other.” Although not of a particularly refined professional vocabulary, this metaphor referred to my wish to explore any possibilities of reducing the extreme level of isolation in interpersonal actions caused by a brain injury to one of the individuals. In considering the human social brain, Cozolino (2006) suggested, “Our ubiquitous use of physical metaphors to describe our inner experiences may also betray the sensory-motor core of both our subjective experience and abstract thought” (p. 190). Though challenging a purely cognitive explanation of human experience, Cozolino (2006) goes on to argue, “In the same way, many believe that human reason is not derived from abstract logic but rather emerges from our bodily experience within our social and physical environments” (p. 190). Many years after the original event of Episode 1, I engaged in a new research study to uncover an additional layer of meaning of Episode 1.

### Arts-based pilot study: “In Visible Hands”

In 2010, I moved from Ireland to Norway to take up a position as associate professor in music therapy at the Grieg Academy and the Grieg Academy Music Therapy Research Centre (GAMUT) at the University of Bergen. Once there, my research continued to extend from the neurobiological foundations of attachment that I had come into contact with thanks to Jane Edwards in Ireland, and to move toward the themes of non-individualized cognition and socioneural ecologies. It was clear from the constructs and categories from my doctoral research and expressive writing that I needed to further explore the relational aspects of Episode 1, not from a psychodynamic and interpersonal understanding of the concept relationship, but from one that focuses on a hypothesis of the social core of intrapersonal processes and extended mind theory in relation to the shared and ecological processes of perception, action, and cognition. In this new theoretical and geographic space, I became interested in a hypothesis that no individual can be authentically and rationally considered without an integration of “the other” in that consideration.

The initial impulse for this new study began after I viewed Episode 1 together with a newly arrived colleague, Dr Jill Halstead. Jill is a musicologist and scholar with extensive knowledge of a wide range of topics, including feminist theory and the domains of music action, creativity, and embodiment. In our initial conversations, Jill and I found out that we share a passion for topics and themes that cross conventional domains, bridging and fusing to create new areas and forms of enquiry. After one discussion, I watched Episode 1 once again and realized that something was missing from the visual material on the video. Although the recorded artifact contained the complete audio scenario, it became clear that a most obvious element of the lived interaction was missing – my body.

Jill had begun introducing me to the work of authors such as Mark Johnson (1990) and Beatrice Allegranti (2011) on embodiment and meaning, and these writings highlighted the absence of my body and, through that, its potential presence. The video camera had captured only Bert's movements and the back of the upright piano, behind which my bodily self was completely hidden. Obviously, my mind had been, up to that point, content with artificially “ghosting” my physical existence back into the scene by acoustic association, rather than by actual visual presence in the video documentation. The very hand that had played the four-phrase melody pattern was missing. There was no finger that played the first note “f” of the melody in Episode 1. My hands were invisible.

As a result of this initial thought in 2011, I began thinking about applying for research funding for a project that would explore the meaning that music therapists gave to their hands in relation to specific moments in their creative music making in their work as a music therapist. The title of the project was chosen to be “In Visible Hands” to signify the bodily site of potential meaning in music therapy work.

Frequently, when I began describing the idea of the project to my colleagues, I noticed myself forming my right hand into the position of the very first few moments of the video footage, perhaps the very first note of Episode 1. Whereby my words in the past research steps were telling the story of those actions, it was my body that showed that story. The narrative was becoming visible through my hand's reenactment of the episode. I felt a confirming sense that the reconstruction of my hand in that episode was the next piece of work in this long-term repeated-immersion process of research.

I began playing with the idea of creating a sculpture of my hand in a stance or position from that episode. I dreamt of using three-dimensional digital scanning and virtual representation. I took multi-angle photographs of my hand and attempted to create classic red–green 3D images that could be viewed with 3D glasses and other technological solutions such as 3D printing or holograms. I also filmed my hand playing the music of the episode on the piano from six different angles with a video camera to create a video reconstruction of my hand's movements in Episode 1.

Finally, I rested with the idea of creating a highly detailed body mold and cast of my hand that would result in a physical restructure or sculpture of my hand with the finest level of detail possible. Convinced by the potency of this process of physical reconstruction, I applied together with Jill for funding for the materials for an arts-based research study to produce between 10 and 12 body molds and casts of hands of music therapists who had worked in a variety of areas related to the use of music in health and well-being. The production of the hand casts was augmented by a semistructured interview during the casting processes, to be followed up with a written interview based on initial analyses of the first verbal interview. The project is in progress at the time of writing and includes the hands of music therapists working in prisons, child and adolescent welfare, adult mental health care, kindergartens, and neurological rehabilitation. It is planned that the project will come to a conclusion in 2014.

In the first steps of the project, I had to learn a great deal about the materials, techniques, and challenges of human body casting, which I managed only with the kind assistance of Germain Ngomo, an artist and educator of body casting and sculpture at the Norwegian Academy for Fine Arts, and Maurice Velterop, an industrial supplier of materials to the fine arts and construction professions. Once skilled, I began with creating my first-ever cast of Jill's hand and then progressed a few days later, on 30 October 2012, to reconstruct my own right hand. I chose the position of playing the first note (“f1”) of the episode with the index finger of my right hand, as captured in the video footage of Episode 1 with Bert.

The process is straightforward. A mix of alginate powder with water is used to create a mold by immersing the hand in the desired position into the mix and waiting for approximately 10 min whilst the mixture becomes firm but flexible enough to remove the hand without damaging the rubber-like mold. I then used two-component acrylic, which was poured into the mold, shaken, tapped, and rolled around to release air bubbles before being left to stand for 1 h to set into a very hard but not brittle, off-white duplication of my hand.

On removing the cast from the mold, I was struck by the lifelike quality of the cast. Every fingerprint, vein, fold, and contour of my hand was present. All of the scars of my biography were glaringly present. A curious inconsistency in the molding mixture led to imperfections in the cast of my little finger, the only finger on that hand that I have ever significantly damaged as a child in sports. This elicited the important consideration of the physicality of the body of the therapist and how this too places restraints or characteristics of improvisation and interaction, infusing the meaning of the body in music improvisation.

Once I was finally sitting with my “hand” in my hands, many thoughts sprinted into my consciousness. Initially, I began to realize that I could begin to contemplate why I had improvised what I had from completely new vantage points. For example, earlier studies on neurofunction and the peripheral nervous system provide a great deal of knowledge about how the index finger and particularly the thumb (which were prominent in Episode 1) are well represented at cortical levels of the brain and how the representation of the hand of musicians develops differently at a neural level than it does for non-musicians (Münte, Altenmüller, & Jäncke, 2002). This accounts for the extreme differentiation of the resolution of movement and overall fine motor abilities of musicians. This may also provide a neurophysiological rationale for the selected action when improvising in a situation that demanded extremes in terms of temporal physical control, immaculate control of the trajectories and dynamics of my music-making body. It became clear through the pilot study that my body may be considered as the main vehicle of relating in that scenario and that it makes sense to make use of those parts of my body that are most extensively trained (I have played the piano since I was 6 years old) in exactly that way.

**Figure 1:**
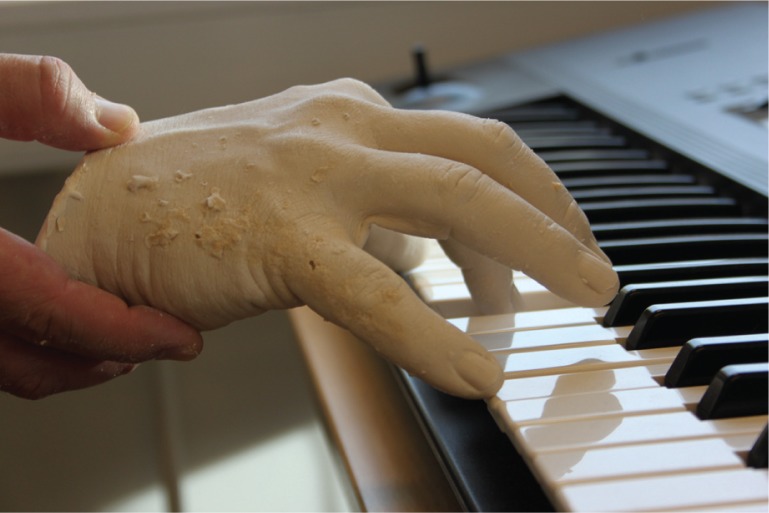
The body cast of the author's hand playing the first note of Episode 1 in the arts-based research project titled “In Visible Hands.”

The sense of touch on the index finger of my dominant (right) hand is my most diversified and biographically significant resource that I may have. Not only in terms of music performance skills on the piano, but also in terms of semantic connectivity in gestures and cognition, it is a primary point of orientation in terms of body scheme. It seems likely that I may have chosen this finger in many occasions during my 11 years of improvising in therapy for such important duties. After all, earlier parts of these research studies hint that it is this finger that is a part of Bert's emergent agency in determining the actions of another person – my perception of Bert's actions determines what my finger does (Figure 1).

On holding the hand cast, I was reminded that my thumb and little finger are just as important as my index finger. The thumb has the largest area of representation in the motor cortex of all fingers. Together with the little finger, a hand form is created that provides the most stable and balanced movement pattern when spread over an octave of piano keys as in the episode. My body, as seen through my right hand, is generating physical stability, contrasting the interpersonal scenario of insecurity, uncertainness, and hypothetical propositions of coordinative attempts of musical expression and communication with Bert. The hand is a part of isolation, and it is a resource of minimal contact that is elementary in the exploration of the ways in which Bert and I are connected or interrelated.

Through putting various perspectives together, it becomes clearer and clearer that I am in fact being led by Bert. He is leading me (without any conventionally obvious signs of consciousness or intentionality about this at that time) through his movements. In fact, my finger movements are both responses to his movements and also provide me with a unique way of perceiving him. Action, in this context, is a perceptive act of intention. Though 19 years have passed, I can today still feel the surface of the piano key of that first note of Episode 1. It was my sense of touch, through my embodied intentionality to communicate, that supported the process of searching for “a way to get in touch” with Bert.

While meaning at the outset was hidden, it has become clearer over time and has shown that some aspects of meaning were present, if not then visible or obvious. By considering the potential meaning held in the visible hand (both real and reconstituted in the body cast or sculpture), potential meaning has become visible. Interestingly, this meaning has been “held” and embodied in my hand over the past 19 years. Fascinatingly, meaning is also embodied in this visible acrylic hand crafted from the fleshy, sensing, and pulsating reality of my being.

## Final thoughts

After studying Episode 1 for many years, I have experienced a shifting and diversification of perspectives on the episode of therapy that has been led by continuing research and immersion, study and learning, and moving toward new people and theories. Through this long-term process, it has become more and more obvious that the conventional ways of analyzing the video material with music transcription and narrative analysis only made it possible to consider what could be easily seen and heard. It was then at this junction that the idea emerged to look for what was missing from the video footage.

### The sense of sense

Whilst reading classic and contemporary texts on the senses (MacPherson, 2011) and human dynamics (as discussed in Stern, 2010), I experienced a twist of my focus onto my own senses and how they may be used to access what was missing from the video footage. Initially, I was most interested in my sense of touch on the piano keys, and this emerged to represent a milestone in the maturation of my research outlook.

Focusing on the sense of touch led me to consider my hand itself, my understanding of theories of embodiment, and the relationships between the central and peripheral, the individual and social. By considering the visible, audible, and embodied material of music therapy, it is possible to consider and reflect on the meaning of our bodies, individual and social. As Aldridge (1996) noted, “[T]he body has a central role in post-modern society. The relationship with the self is with the body; it is here that we have the interface of internal and external. How we experience the unfolding of our experience is reflected in our bodies” (p. 110). Although bodily reflection of experience is undoubtedly significant, the research reported in this article highlights ways in which a relationship with the “other” occurs within, and can be extracted through, our bodies. This acknowledgment of the body's significance should not be restricted to the bodies of clients or patients. Music therapists are encouraged to consider their own bodies in relation to the bodies, minds, and social roles that we choose or are given by others. We may consider music in therapy at a cellular or community level and all resolutions in between. This perspective is endorsed by Cozolino (2006) in his eloquent portrayal:

If we use Mother Nature as a guide, we see that when she likes an idea, she tends to stick with it, and she does so by conserving structures and strategies through increasing layers of complexity. Assuming this is true (which I do), we stand to learn a great deal from zooming in and out, from “neurons to neighborhoods,” while resisting the urge to become attached to any particular frame of reference. In this way, we may gain a deeper understanding of the interwoven tapestry of biological, psychological, and social processes that comprise human life. (Cozolino, 2006, pp. 3, 4)

### Reconceptualizing traumatic brain injury: traumatic social brain injury

As described at the outset of this article, traumatic brain injury is conventionally described in terms of the person injured. Unfortunately, however, this has serious consequences in terms of the limited conceptualization of the nature of traumatic brain injury. Although the individual must be considered, this should never be in isolation and it is the damage to the access and integration of the ecological context of the patient that is of consequence for their care and potential of rehabilitation. This demands a perhaps reluctant, but necessary, reconsideration of the nature of traumatic brain injury and indeed the fundamental conceptualization of the brain, body, and ecological context in everyday life.

One way of achieving this is to consider not only the brain or central nervous system but also the peripheral nervous system both in terms of the role it plays in responding to impulses for movement and as a perceptive system that is capable of directing the work of the central nervous system. Thus, the brain may be considered to be just one part (albeit a highly important one) of the systems of action and perception and should not be considered as the sole site of meaning and sense of being. This is a stance taken in recent considerations of embodied cognition (Shapiro, 2011), extended cognition (Menary, 2010), and social neuroscience (Cacioppo, Visser, & Pickett, 2006). If we then add to this scenario not just one individual but also a second one, a partner within a human ecological scenario, we may be compelled to consider the central and peripheral nervous systems of both individuals. An action and perception scenario becomes at once more interesting and messy. In fact, to generate this perspective, we need only to acknowledge that we are naturally social beings and that

the individual neuron or a single human brain does not exist in nature. … Thus, understanding the brain requires knowledge of the healthy, living brain embedded within a community of other brains: Relationships are our natural habitat. (Cozolino, 2006, p. 11)

### Long-term repeated immersion: what the case study of traumatic brain injury highlights for qualitative research in the future

As this research process has shown, traumatic brain injury is characterized by extremes of isolation and idiosyncratic needs and most severe social devastation. The experience of severe traumatic brain injury is unimaginable, a prime example of the nature of destructive plasticity (Malabou, 2012) that changes lives beyond the boundaries of what we are commonly prepared to believe. Through long-term repeated-immersion, the multiple perspectives generated by the use of multiple and different qualitative research methods have shown that music therapy contributes to a reduction of isolation and an increase in the opportunity for the injured person and the music therapist to connect and relate. This epistemological commitment to the use of multiple methods and long-term immersion has clearly identified the need for a consideration of an innovative model of music therapy that considers the interrelated and socioplastic nature of injury and rehabilitation, a worthy work agenda for the future beyond the scope of this article. Although this exploration of meaning in music improvisation for people affected by traumatic brain injury began many years ago, it may be that innovative understandings not only are to be found in our brains or recorded artifacts, but also may lie in our very own hands, whether analogic or fleshy.
